# Cable Interlayer Slip Damage Identification Based on the Derivatives of Eigenparameters

**DOI:** 10.3390/s18124456

**Published:** 2018-12-16

**Authors:** Jintu Zhong, Quansheng Yan, Liu Mei, Xijun Ye, Jie Wu

**Affiliations:** 1 School of Civil Engineering and Transportation, South China University of Technology, Guangzhou 510641, China; zhongjintu@gmail.com (J.Z.); cvqshyan@scut.edu.cn (Q.Y.); wujiemc@gmail.com (J.W.); 2 Guangdong Provincial Key Laboratory of Durability for Marine Civil Engineering, Shenzhen University, Shenzhen 518060, China; 3 School of Civil Engineering, Guangzhou University, Guangzhou 510006, China; xijun_ye@gzhu.edu.cn

**Keywords:** damage identification, cable interlayer slip, eigenpair sensitivity method, discrete system, Tikhonov regularization

## Abstract

Cables are the main load-bearing structural components of long-span bridges, such as suspension bridges and cable-stayed bridges. When relative slip occurs among the wires in a cable, the local bending stiffness of the cable will significantly decrease, and the cable enters a local interlayer slip damage state. The decrease in the local bending stiffness caused by the local interlayer slip damage to the cable is symmetric or approximately symmetric for multiple elements at both the fixed end and the external load position. An eigenpair sensitivity identification method is introduced in this study to identify the interlayer slip damage to the cable. First, an eigenparameter sensitivity calculation formula is deduced. Second, the cable is discretized as a mass-spring-damping structural system considering stiffness and damping, and the magnitude of the cable interlayer slip damage is simulated based on the degree of stiffness reduction. The Tikhonov regularization method is introduced to solve the damage identification equation of the inverse problem, and artificial white noise is introduced to evaluate the robustness of the method to noise. Numerical examples of stayed cables are investigated to illustrate the efficiency and accuracy of the method proposed in this study.

## 1. Introduction

In recent decades, bridges with cable systems, such as cable-stayed bridges and suspension bridges, have been increasingly used with the rapid development of sea-crossing bridges in the global transportation field. The structure of a cable system bridge can experience relative slip damage between the steel wires in cables when subjected to lateral external loads. Then, the local bending stiffness of the cable is significantly reduced in the interlayer slippage state, which affects the mechanical performance of the cable [1,2,3]. Cables are the main load-bearing structural components of long-span bridges, and the physical properties of cables play a key role in structural health. As interlayer relative slip between cable wires repeatedly occurs during bridge operation, the cable will inevitably experience fretting wear during its life cycle, thereby reducing the service life of the cable. The most common cable structure of a long-span bridge involves parallel steel-strand cables. The relative slip properties of parallel steel-strand cables are equivalent to those modeled by the interlayer slip of a parallel laminated beam.

**Figure 1:**
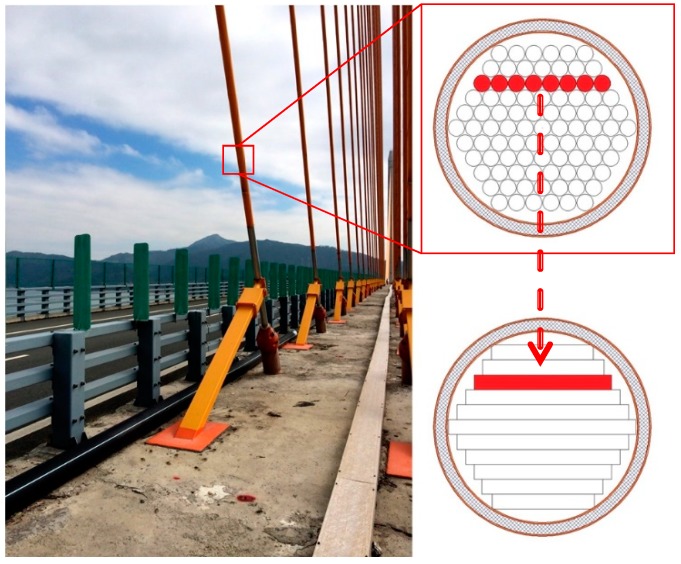
Parallel steel cable and the equivalent laminated beam.

The cable section shown in Figure 1 is a common parallel steel-strand cable from a long-span cross-sea cable-stayed bridge in China. When the parallel cable is subjected to a lateral force, the layered wires shown in red in Figure 1 experience deformation, and there is almost no relative movement among the wires in the layer. This feature is similar to that of a frictional laminated beam subjected to a vertical external load. Therefore, ignoring the action of the outer rubber protective layer of the stay cable, the illustrated parallel cable section can be considered equivalent to a multi-laminated beam model. In this paper, the cable is equivalent to a frictional laminated beam with a uniform bending moment of inertia.

Yan et al. [4] compared the main cable test results and actual measured values for a long-span suspension bridge in China and found that the cable wire interlayer slip caused secondary stress, which affected the deflection of the cable. Campi and Monetto [5] studied the dynamic performance of the interlayer slip of two-layer laminated beams and found that the interlayer slip damage reduced the stiffness coefficient of the stacked beams. Monetto [6] further extended the study to the dynamic properties to three-layer laminated beams and obtained a dynamic analytical solution for a laminated beam affected by interlayer slip damage.

Many methods have been used to detect damage to laminated beams or other bridge structures. Cao and Qiao [7] used the progressive wavelet transform method and successfully detected crack damage in laminated beams. Rucevskis et al. [8] identified the damage to a laminated beam by extracting mode shape information obtained from vibration experiments and observed the loss of local stiffness in the structure. Zhang et al. [9] proposed an undamaged method of microdamage detection based on a strain model method and effectively located the damage position by comparing the strain-modal curves obtained from dynamic tests of the damaged structures. Wang et al. [10] proposed a new damage detection method based on the conception of the inner product vector (IPV) and theoretically analyzed the robustness to measurement noise of the proposed method. Zhou et al. [11] proposed a new approach for detecting long-term structural damage using transmissibility combined with hierarchical clustering and similarity analysis. This method can avoid setting a baseline according to prior distance measure-based damage detection procedures and is more effective than the Hausedorff distance-based and Euclidean distance-based damage detection procedures. However, few studies have focused on effectively identifying cable interlayer slip damage.

Eigenvalue sensitivity and eigenvector sensitivity have also been used in structural damage detection based on modal parameters. Wu and Law [12] studied the damage to a frame structure by decomposing the system matrices into static eigenvalues and eigenvectors. Qiu et al. [13] improved the eigenvalue sensitivity analysis method by linearly substituting the undamaged structure mode shapes with changed structure mode shapes and obtained high accuracy in damage location and identification. Dilena and Morassi [14] identified isolated damage in a discrete mass-spring beam-like system with changes in eigenvalues and eigenvectors in the structural system. The effective calculation of eigenparameter derivatives is important in detecting the effect of parameter variations on the dynamic behavior of a structure. Like the finite element method, it is feasible to approximate the dynamics of a continuous structure based on the dynamics of a discrete structural system. Qin et al. [15] introduced the particle swarm optimization algorithm and combined high-order eigenvalue parameters to update a bridge structure model. The results showed that the high-order vibration modes can be used to accurately estimate and predict the structural response and structure health. Sensitivity analysis is a reliable method of structural analysis. Cao et al. [16] even combined sensitivity analysis and the Jacobian matrix to perform an iterative form-finding analysis for the main cable of a suspension bridge.

An eigenpair sensitivity method for a discrete structural system is introduced in this study to identify the interlayer slip damage. In Section 2, we deduce the relationship between interlayer slip and the dynamic vibration function of a laminated beam. A method for calculating the eigenpair derivatives of the discrete system and the damage identification algorithm are developed in Section 3. Two numerical examples are given in Section 4 to illustrate the efficiency and accuracy of the identification method proposed in this study. Finally, conclusions are drawn in Section 5.

## 2. Interlayer Slip Damage in a Cable Structure

According to the interlayer slip properties of a frictional laminated beam [17], the interlayer slip causes the laminated beam to be divided into many subsections in the cross-sectional direction, and there is a dynamic frictional force at each contact face. To study the influence of the local interlayer slip on the overall vibration of the laminated beam, it is assumed that the slip damage occurs uniformly in the entire unit for a laminated beam of unit length. The slip state of the laminated beam is defined according to the following situations. (1) When interlayer slippage first occurs, the laminated beam is in the initial interlayer slip state, denoted as $*|{}_{1-slip}$. (2) When interlayer slippage occurs j times, the laminated beam is in the *j*th interlayer slip state, denoted as $*|{}_{j-slip}$. (3) When all the contact layers of the laminated beam have slipped, the laminated beam is said to be in the full slip state, denoted as $*|{}_{all-slip}$.

**Figure 2:**
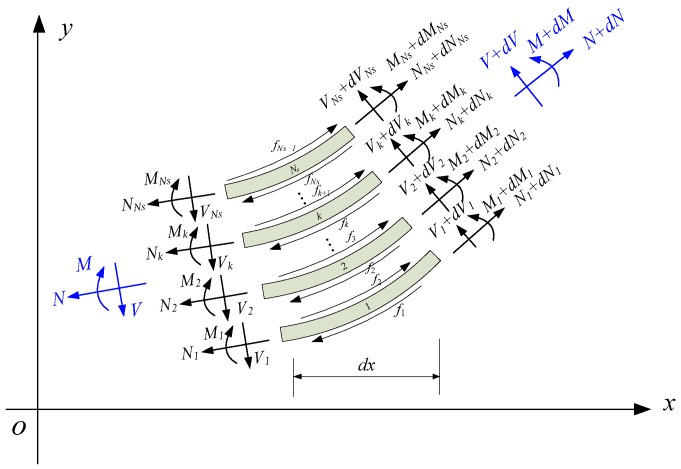
Free-body diagram of the slip section of a laminated beam.

We assume that the cross-section of a laminated beam is divided into $N_{s}$ subsections under the action of interlayer slip, as shown in Figure 2, wherein the internal forces acting on the *k*th subsection include the axial force $N_{k}$, bending moment $M_{k}$ and shear force $V_{k}$. The upper and lower contact layers of the segment are subjected to interlayer frictional forces acting in opposite directions, denoted as $f_{k},f_{k+1}$. Neglecting the influence of the rotational moment of inertia, the bending moment balance equation for each subsection can be expressed as follows:

(1) $$\{\begin{array}{c} dM_{1}+V_{1}dx-\left(f_{1}+f_{2}\right)\frac{h_{1}}{2}dx=0\\ dM_{2}+V_{2}dx-\left(f_{2}+f_{3}\right)\frac{h_{2}}{2}dx=0\\ \cdots \\ dM_{k}+V_{k}dx-\left(f_{k}+f_{k+1}\right)\frac{h_{k}}{2}dx=0\\ \cdots \\ dM_{N_{s}}+V_{N_{s}}dx-\left(f_{N_{s}}+f_{N_{s}+1}\right)\frac{h_{N_{s}}}{2}dx=0 \end{array}$$

where $h_{k}$ is the high of the cross-section of each subsection.

The superposition of all the fractional equations in Equation (1) can be written as follows.

(2) $$\sum_{k=1}^{N_{s}}dM_{k}+\sum_{k=1}^{N_{s}}V_{k}dx-\sum_{k=1}^{N_{s}}\left(f_{k}+f_{k+1}\right)\frac{h_{k}}{2}dx=0$$

The bending moment of the *k*th subsection can be formulated as follows:

(3) $$M_{k}=EI_{i}^{p}|{}_{j-slip}\omega^{\prime \prime }(x)$$

where $I_{i}^{p}|{}_{j-slip}$ is the partial moment of inertia in the *j*th slip state and ω(x) is the vertical displacement of the laminated beam.

The shearing force and axial force between subsections and the entire laminated beam satisfy the following equations.

(4) $$V=\sum_{k=1}^{N_{s}}V_{k},N=\sum_{k=1}^{N_{s}}N_{k}$$

Substituting Equations (3) and (4) into Equation (2), and continuing to differentiate twice with respect to length *x*, we get:

(5) $$\sum_{k=1}^{N_{s}}EI_{i}^{p}|{}_{j-slip}\frac{\partial^{4}\omega }{\partial x^{4}}+\frac{\partial V}{\partial x}-\frac{\partial }{\partial x}\left(\sum_{k=1}^{N_{s}}\left(f_{k}+f_{k+1}\right)\frac{h_{k}}{2}\right)=0$$

Because the interlaminar friction is assumed to be Coulomb friction, the friction is also fixed when the slip state is constant. Therefore, the final derivative of the frictional force on the left side of Equation (5) is equal to zero.

The vibration equation of the entire laminated beam is written as follows:

(6) $$\frac{\partial V}{\partial x}+\frac{\partial }{\partial x}\left(N\frac{\partial \omega }{\partial x}\right)+p\left(x\right)=m\ddot{\omega }$$

where p(x) is the uniformly distributed load on the laminated beam, the unit of p(x) is N/m. m is the mass of the laminated beam of unit length, the unit of m is kg/m.

Equations (5) and (6) can be combined to obtain the vibration equation of the laminated beam in the slip state by considering the damping C:

(7) $$m\ddot{\omega }+B_{s}\frac{\partial^{4}\omega }{\partial x^{4}}+C\dot{\omega }-N\frac{\partial^{2}\omega }{\partial x^{2}}=p\left(x\right)$$

where $B_{s}=\sum_{k=1}^{N_{s}}EI_{i}^{p}|{}_{j-slip}$ is the overall bending stiffness of the laminated beam in the slip state.

Equation (7) indicates that the vibration of the frictional laminated beam in the interlayer slip state is consistent with the vibration of a Euler-Bernoulli beam, which is generally subjected to an axial force. However, the stiffness of the slipped partial laminated beam changes in different slip states. Therefore, the interlayer slip damage of the frictional laminated beam can be identified by the stiffness variation of local elements. Thus, it is reasonable to treat the cable as a discrete mass-spring system for slip damage identification.

## 3. Theory of Interlayer Slip Damage Identification

### 3.1. Mass-Spring Systems

**Figure 3:**
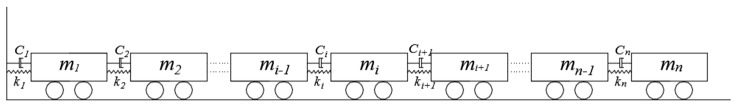
Discrete mass-spring system.

A mass-spring-damper system consists of n masses *m_i_* (*i* = 1, 2, ..., *n*) that are consecutively connected by linear elastic springs *k_i_* (*i* = 1, 2, ..., *n*) and dampers *c_i_* (*i* = 1, 2, ..., *n*), as shown in Figure 3.

The matrix associated with the dynamic equation of a discrete structural system can be expressed as follows:

(8) $$M\ddot{u}+C\dot{u}+Ku=F(t)$$

where $u,\dot{u},\ddot{u}$ are the displacement, velocity and acceleration response vectors of the discrete structure, respectively; F(t) is the vector of applied forces; and M is a symmetrical matrix with mass values along the diagonal. *K* and *C* are tri-diagonal positive semi-definite matrices that can be expressed as follows.

$$\begin{array}{l} K=\left(\begin{array}{cccccc} k_{1}+k_{2} & -k_{2} & 0 & \cdots & 0 & 0\\ -k_{2} & k_{2}+k_{3} & -k_{3} & \cdots & 0 & 0\\ 0 & -k_{3} & k_{3}+k_{4} & \cdots & 0 & 0\\ \vdots & \vdots & \vdots & \ddots & \vdots & \vdots \\ 0 & 0 & 0 & \cdots & k_{n-1}+k_{n} & -k_{n}\\ 0 & 0 & 0 & \cdots & -k_{n} & k_{n} \end{array}\right) \end{array}$$

$$C=\left(\begin{array}{cccccc} c_{1}+c_{2} & -c_{2} & 0 & \cdots & 0 & 0\\ -c_{2} & c_{2}+c_{3} & -c_{3} & \cdots & 0 & 0\\ 0 & -c_{3} & c_{3}+c_{4} & \cdots & 0 & 0\\ \vdots & \vdots & \vdots & \ddots & \vdots & \vdots \\ 0 & 0 & 0 & \cdots & c_{n-1}+c_{n} & -c_{n}\\ 0 & 0 & 0 & \cdots & -c_{n} & c_{n} \end{array}\right)$$

The state vector $x=\left\{\begin{array}{c} u\\ \dot{u} \end{array}\right\}$ can be introduced to reduce the order of Equation (8) as follows.

(9) $$\dot{x}=Ax+BF(t)$$

where $A=\left[\begin{array}{cc} 0_{n\times n} & I_{n\times n}\\ -M^{-1}K & -M^{-1}C \end{array}\right]B=\left[\begin{array}{c} 0_{n\times 1}\\ M^{-1} \end{array}\right]$

### 3.2. The Eigenparameter Sensitivity Method

System features, such as eigenvalue and eigenvector parameters, can represent the dynamics characteristic of the system. Changes in the variables associated with system dynamics can cause changes in the system characteristic parameters. In this case, the eigenparameter sensitivity method is introduced to identify the interlayer slip damage.

The eigenvalues are assumed to be distinct [18], and the characteristic equation of matrix **A** corresponding to the *j*th order eigenpair $\left\{\lambda_{j},\phi_{j}\right\}$ is as follows.

(10) $$A\phi_{j}=\lambda_{j}\phi_{j}$$

It is assumed that when the structure is damaged, a certain physical parameter *α* changes, and the amount of change is Δ*α*. By differentiating Equation (10) with respect to parameter *α*, the following equation can be obtained.

(11) $$\frac{\partial A}{\partial \alpha }\phi_{j}+A\frac{\partial \phi_{j}}{\partial \alpha }=\lambda_{j}\frac{\partial \phi_{j}}{\partial \alpha }+\frac{\partial \lambda_{j}}{\partial \alpha }\phi_{j}$$

Based on the normalization condition, for proportionally damped systems

(12) $$\phi_{j}{}^{H}W\frac{\partial \phi_{j}}{\partial \alpha }=0$$

where **W** is a weighting matrix, which can be taken as a unit matrix.

The detailed proof for Equation (12) can be found in Reference [18].

Combining Equation (11) and (12) yields the following relation.

(13) $$\left[\begin{array}{cc} A-\lambda_{j}I & -\phi_{j}\\ \phi_{j}{}^{H}W & 0 \end{array}\right]\left\{\begin{array}{c} \frac{\partial \phi_{j}}{\partial \alpha }\\ \frac{\partial \lambda_{j}}{\partial \alpha } \end{array}\right\}=\left\{\begin{array}{c} -\frac{\partial A}{\partial \alpha }\phi_{j}\\ 0 \end{array}\right\}$$

The first-order eigenpair derivatives can be obtained from Equation (13).

(14) $$\left\{\begin{array}{c} \frac{\partial \phi_{j}}{\partial \alpha }\\ \frac{\partial \lambda_{j}}{\partial \alpha } \end{array}\right\}={\left[\begin{array}{cc} A-\lambda_{j}I & -\phi_{j}\\ \phi_{j}{}^{H}W & 0 \end{array}\right]}^{-1}\left\{\begin{array}{c} -\frac{\partial A}{\partial \alpha }\phi_{j}\\ 0 \end{array}\right\}$$

### 3.3. The Identification Problem

When a structure is in a damaged working state, there is a difference between the measured response and the predicted response of the structure. The damage identification problem of the structure can be regarded as a mathematical problem that involves optimizing or minimizing the objective function of the measured structural response and the predicted response.

The eigenpair sensitivity of a discrete structural system can be determined based on forward analysis. Therefore, the damage identification problem based on the eigenpair sensitivity method can be transformed into an inverse optimization problem with modal test data. The degree of damage to the system is obtained by adjusting the structural parameters, such that the predicted structural response is closer to the measured response. In other words, the damage identification problem for a discrete structural system is equivalent to finding a suitable physical damage parameter ***α*** that yields calculated values that are close to the measured values.

The penalty function method is generally used for modal sensitivity with a truncated Taylor series expansion in terms of the unknown parameters. This expansion is often limited to the first two terms, to produce the linear approximation. The updated parameter value is obtained by minimizing the penalty function; it is an iterative process [19]. According to the penalty function method, the damage identification equation can be expressed as follows:

(15) SΔα=ΔE

where Δα is the variation in the damage parameter of the structure and S is the two-dimensional sensitivity matrix.

(16) $$S=\left|\begin{array}{cccc} \frac{\partial \phi_{1}}{\partial \alpha_{1}} & \frac{\partial \phi_{1}}{\partial \alpha_{2}} & \cdots & \frac{\partial \phi_{1}}{\partial \alpha_{m}}\\ \frac{\partial \lambda_{1}}{\partial \alpha_{1}} & \frac{\partial \lambda_{1}}{\partial \alpha_{2}} & \cdots & \frac{\partial \lambda_{1}}{\partial \alpha_{m}}\\ \cdots & \cdots & \cdots & \cdots \\ \frac{\partial \phi_{n}}{\partial \alpha_{1}} & \frac{\partial \phi_{n}}{\partial \alpha_{2}} & \cdots & \frac{\partial \phi_{n}}{\partial \alpha_{m}}\\ \frac{\partial \lambda_{n}}{\partial \alpha_{1}} & \frac{\partial \lambda_{n}}{\partial \alpha_{2}} & \cdots & \frac{\partial \lambda_{n}}{\partial \alpha_{m}} \end{array}\right|$$

where the subscript *m* of *α* denotes the total number of the damaged parameter *α* in the structure. *m* is equal to the element number of the structure. The subscript *n* of ϕ and λ denotes the order number of the eigenpair.

ΔE is the error vector for the measured data and can be obtained by the following equation.

(17) $$\Delta E=\left\{\begin{array}{c} \phi_{1}\\ \lambda_{1}\\ \vdots \\ \phi_{n}\\ \lambda_{n} \end{array}\right\}-\left\{\begin{array}{c} \bar{\phi_{1}}\\ \bar{\lambda_{1}}\\ \vdots \\ \bar{\phi_{n}}\\ \bar{\lambda_{n}} \end{array}\right\}=\left\{\begin{array}{c} \Delta \phi_{1}\\ \Delta \lambda_{1}\\ \vdots \\ \Delta \phi_{n}\\ \Delta \lambda_{n} \end{array}\right\}$$

$\phi_{n}$ denotes the simulated experimental measured data; $\bar{\phi_{n}}$ denotes the calculated data.

Thus, the damage identification problem involves solving Equation (15) to find Δ*α*. In the least squares method, the solution of Equation (15) can be expressed as follows.

(18) $$\Delta \alpha ={\left[S^{T}S\right]}^{-1}S^{T}\Delta E$$

Because the eigenpair sensitivity matrix ***S*** has a very large matrix condition number, Equation (18) is an ill-conditioned problem. The Tikhonov regularization method is introduced here to obtain a more accurate solution [20]. In the Tikhonov regularization method, Equation (18) can be transformed in the following form by introducing a regularization parameter γ that can eliminate the singularity of the ill-conditioned matrix.

(19) $$\Delta \alpha ={\left[S^{T}S+\gamma I\right]}^{-1}S^{T}\Delta E$$

The Tikhonov regularization parameter γ is also called the non-negative damping coefficient. The solution to Equation (19) is equivalent to minimizing the function J(Δα,γ):

(20) $$\mathrm{argmin}\;J(\Delta \alpha ,\gamma )=\arg\underset{\Delta \alpha ,\gamma }{\min}{\left\|S\Delta \alpha -\Delta E\right\|}^{2}+\gamma {\left\|\Gamma \Delta \alpha \right\|}^{2}$$

where Γ is the Tikhonov choice matrix, which is equivalent to the unit matrix I.

Then, the minimized value of function J(Δα,γ) can be calculated by determining the residual norm ‖SΔα−ΔE‖, the regularization parameter γ and solution norm ‖Δα‖. When the parameter γ approaches zero, the solution approaches that obtained from the least squares method. The optimal Tikhonov regularization parameter γ can be obtained using the L-curve method [21].

The interlayer slip damage parameter *α* is determined by iterative calculations. The updated parameter *α*_(*i*+1)th_ of the *i*th iteration can be calculated as follows.

(21) $$\alpha_{(i+1)th}=\alpha_{ith}+\Delta \alpha_{ith}$$

The calculation converges when the following criterion is met:

(22) $$\frac{\left\|\alpha_{(i+1)th}-\alpha_{ith}\right\|}{\left\|\alpha_{ith}\right\|}\leq Tol$$

where Tol is the tolerance of damage identification.

The main calculation steps in the interlayer slip damage identification algorithm are as follows.

(1) Calculate the eigenpair sensitivity matrix of the structural system using Equation (14).
(2) Calculate the error vector ΔE based on Equation (17).
(3) Calculate the variation in the damage parameter Δ*α* using the Tikhonov regularization method.
(4) Update the parameter *α*_(*i*+1)th_ of the *i*th iteration with Equation (21).
(5) Perform iterative calculations until the conditional convergence criterion is satisfied for iteration termination.

### 3.4. Robustness of Artificial Measurement Noise

To assess the robustness of the damage identification algorithm in this paper, noise perturbation is performed by adding artificial measurement noise to the data [22]:

(23) $$\phi_{noise,ij}=\phi_{ij}+E_{p}\cdot N_{noise}\cdot \sigma \left(\phi_{ij}\right)$$

where $\phi_{noise,ij}$ and $\phi_{ij}$ are the mode shape components of the *j*th mode with i degrees of freedom with noise and without noise, respectively; $E_{p}$ is the noise level (in percent); $N_{noise}$ is a standard normal distribution with zero mean and unit standard deviation; and $\sigma \left(\phi_{ij}\right)$ is the standard deviation of the calculated acceleration response.

## 4. Numerical Examples

### 4.1. Example 1: A Mass-Spring-Damper System with 7 DOFs

**Figure 4:**
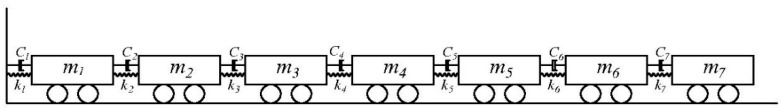
7-DOF mass-spring-damper system.

To verify the validity of the eigenpair sensitivity damage identification method for discrete structural systems, a mass-spring-damper system with 7 degrees of freedom (DOFs) was studied, as shown in Figure 4. The parameters of the system are assumed as $m_{i}=10\mathrm{kg},$$k_{i}=3\times 10^{5}N/m,$$c_{i}=0.5Ns/m,(i=1,2,\ldots ,7)$. The physical quantity of structural damage is represented by the changes in the stiffness parameter and the damping parameter, and the remaining parameters are unchanged.

Case 1: Singular damage to a structure

**Figure 5:**
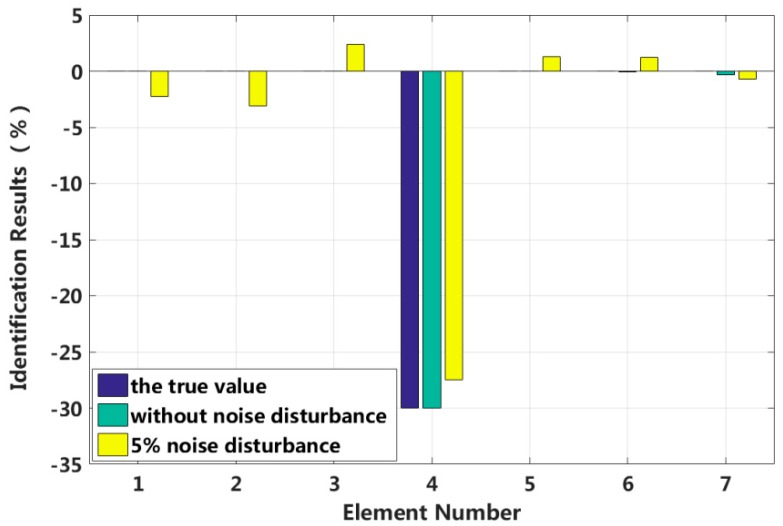
Stiffness identification results for singular damage.

**Figure 6:**
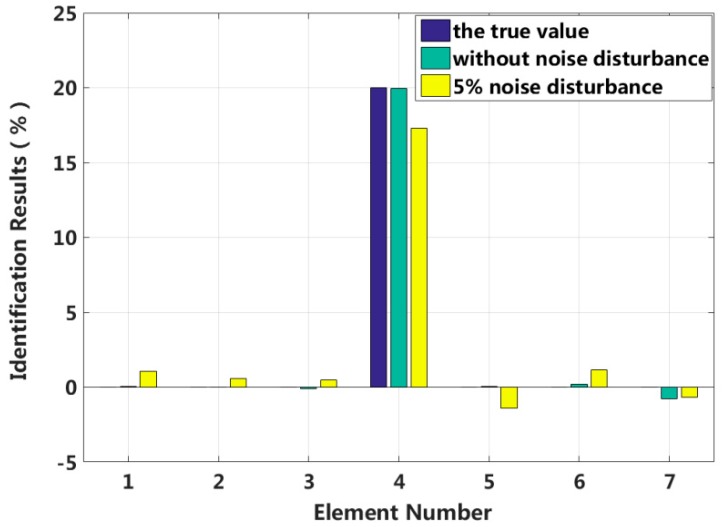
Damping identification results for singular damage.

**Figure 7:**
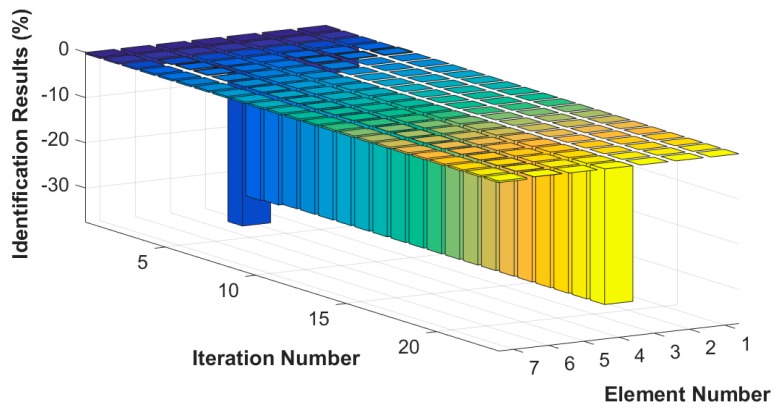
Stiffness damage identification results for each iteration.

**Figure 8:**
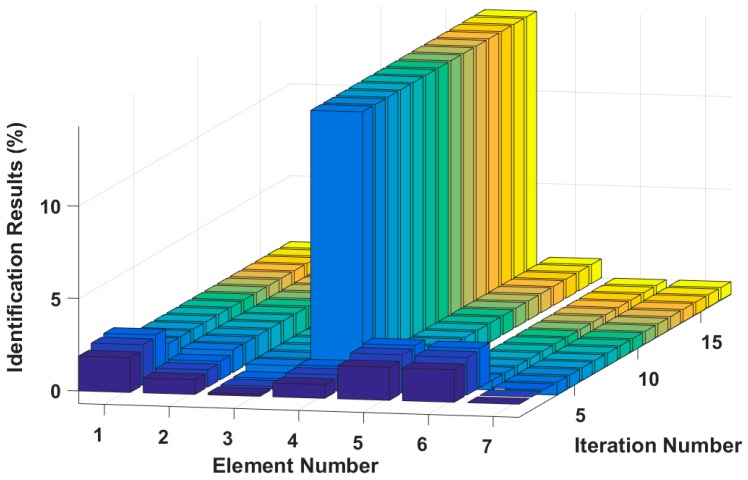
Damping damage identification results for each iteration.

Assuming that the fourth element of the system is damaged, the stiffness $k_{4}$ is reduced by 30%, or the damping $c_{4}$ increases by 20%. The results of stiffness and damping damage identification are plotted in Figure 5 and Figure 6, and the changes in the stiffness and damping results over a series of cyclic iterations are shown in Figure 7 and Figure 8.

Table 1 shows that the eigenpair sensitivity method can accurately locate the position of a damaged element in the structure for the singular damage case with a 5% artificial measurement noise disturbance. Additionally, the damage parameter can be obtained with high precision.

Case 2: Multiple instances of small damage to a structure

**Figure 9:**
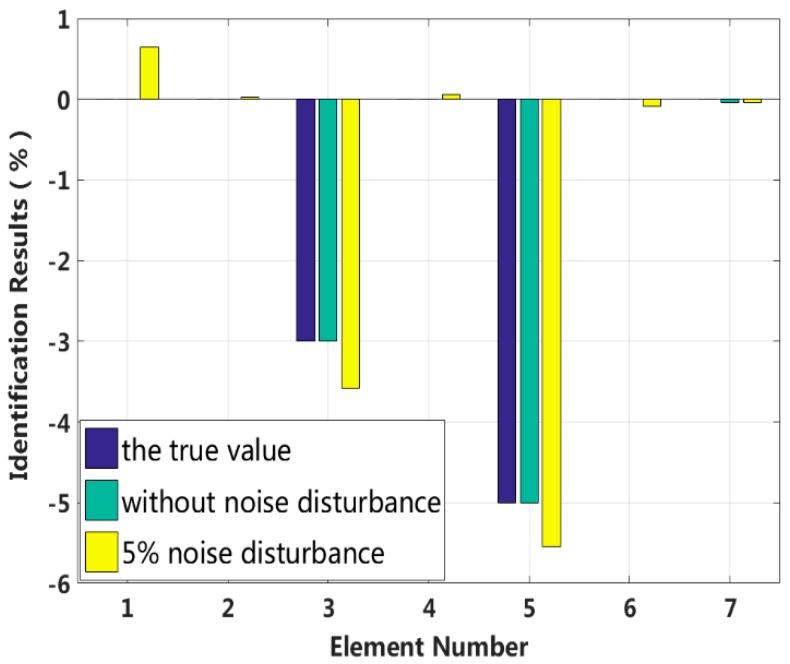
Stiffness identification results for multiple instances of small damage.

**Figure 10:**
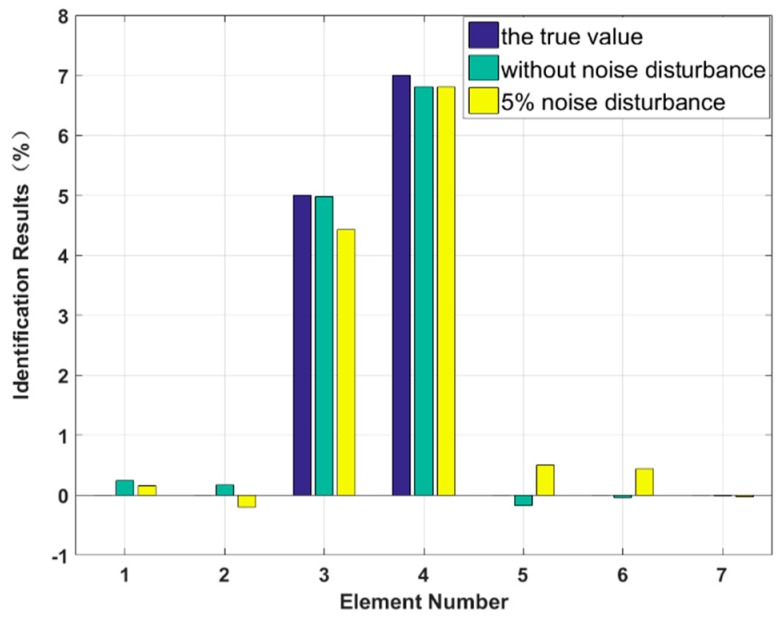
Damping identification results for multiple instances of small damage.

**Figure 11:**
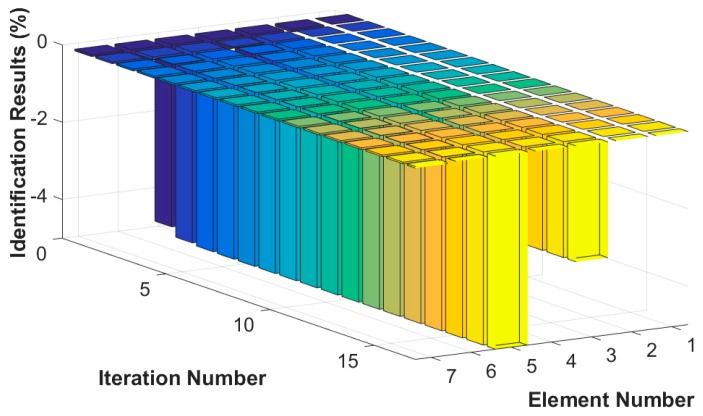
Stiffness damage identification results for each iteration.

**Figure 12:**
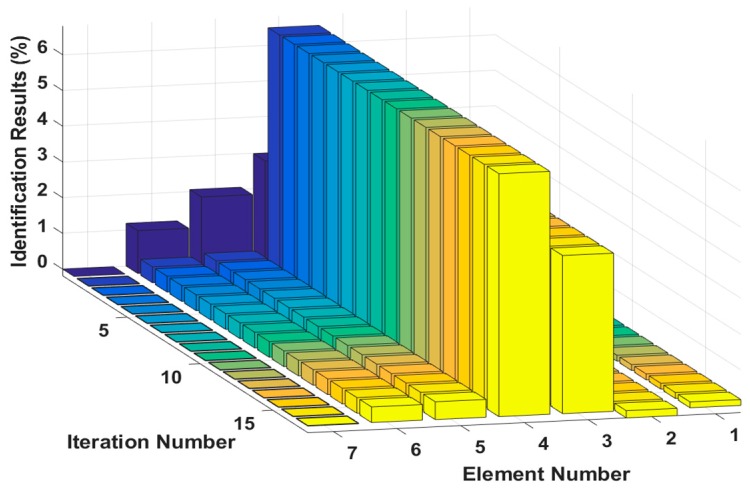
Damping damage identification results for each iteration.

In this case, we assume that two elements are damaged at the same time and that the degree of damage is small. The stiffness of the third and fifth elements, $k_{3}$ and $k_{5}$, are reduced by 3% and 5%, and the damping values of the third and fourth elements, $c_{3}$ and $c_{4}$, are increased by 5% and 7%, respectively. The proposed method is used to identify the multiple small instances of damage, and the results are shown in Figure 9 and Figure 10. The identification errors of the damaged elements with the 5% level of measurement noise disturbance are less than 0.58%. The stiffness and damping damage identification results of the undamaged elements are determined to have identification errors of less than 0.65% and 0.50%, respectively, at the 5% level of measurement noise disturbance. However, the method can effectively eliminate the undamaged elements by comparing the corresponding values to those of damaged elements. The identification results for each iteration are presented in Figure 11 and Figure 12. Both the stiffness and damping parameters are accurately identified after approximately 17 iterations using the proposed method. In small degree of multiple damage case, the proposed method can identify the damage parameter successfully no matter the two damaged elements are consecutive or inconsecutive.

Case 3: Multiple instances of large damage to a structure

**Figure 13:**
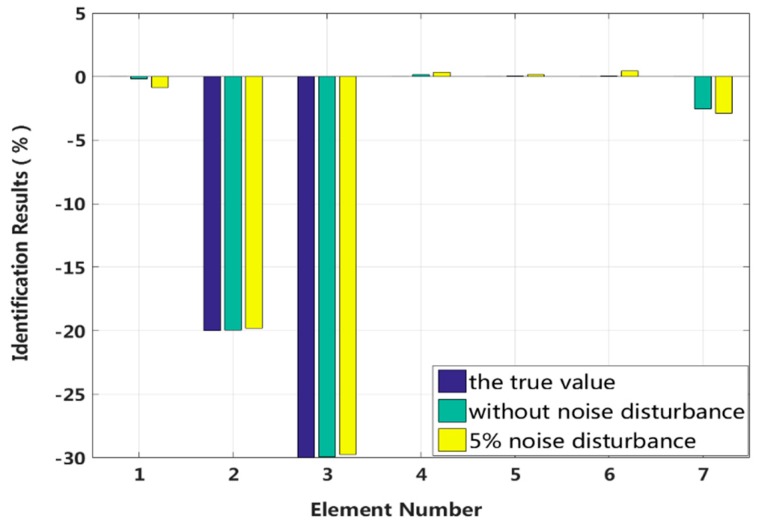
Stiffness identification results for multiple instances of large damage.

**Figure 14:**
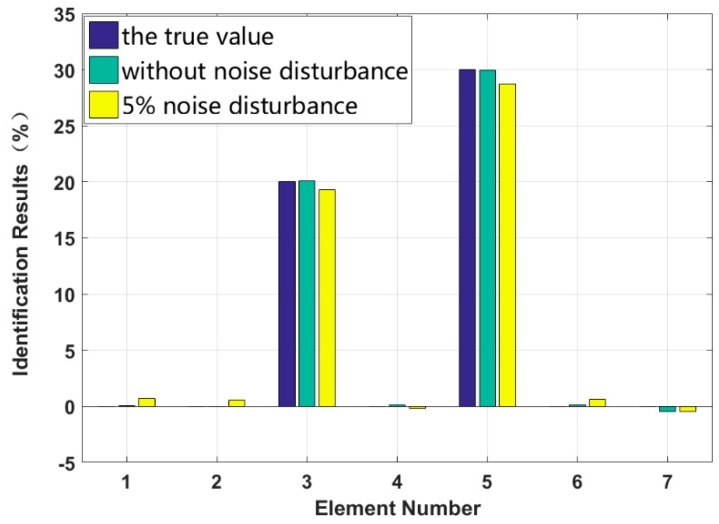
Damping identification results for multiple instances of large damage.

**Figure 15:**
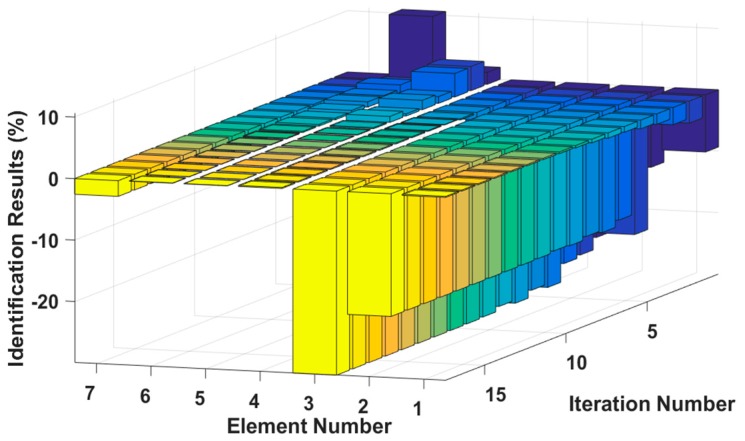
Stiffness damage identification results for each iteration.

**Figure 16:**
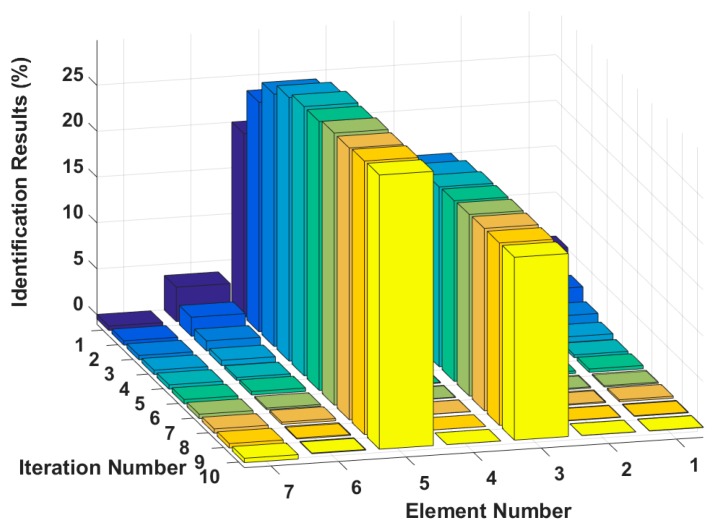
Damping damage identification results for each iteration.

It is assumed that large damage to the second, third and fifth elements occurs, with the degrees of stiffness $k_{2}$ and $k_{3}$ and damping $c_{3}$ and $c_{5}$ perturbed by −20%, −30%, +20% and +30%. The identification results are shown in Figure 13 and Figure 14. The stiffness and damping identification errors of the damaged elements with a 5% level of measurement noise disturbance are less than 0.26% and 1.28%. The stiffness and damping damage identification results of the undamaged elements are determined to have identification errors of less than 2.87% and 0.68%, respectively, at the 5% level of measurement noise disturbance. Figure 15 and Figure 16 are the identification results for each iteration in case 3. A comparison with the iteration figures in the previous case indicates that the proposed method can identify large instances of damage faster than small instances of damage. In large degree of multiple damage case, the proposed method can also identify the damage parameter successfully no matter the two damaged elements are consecutive or inconsecutive. However, if the two damaged elements are consecutive, the identification errors of the undamaged elements are bigger, and higher-order eigenparameters will be needed in the calculation process.

Case 4: Robustness to measurement noise

**Figure 17:**
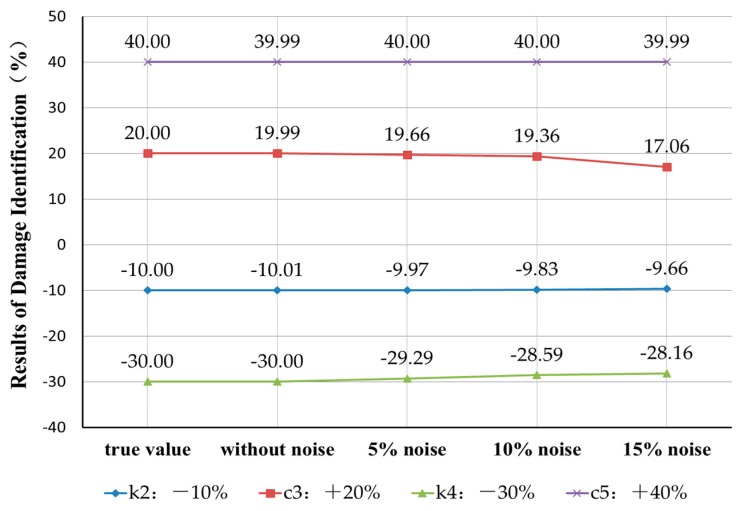
Measurement noise robustness of damage identification based on the eigenpair sensitivity method.

It is assumed that four instances of damage occur to the second, third, fourth and fifth elements with parameters $k_{2}$, $c_{3}$, $k_{4}$ and $c_{5}$ perturbed by −10%, +20%, −30% and +40%, respectively. There are four different artificial measurement noise disturbance levels. The identification results are shown in Figure 17**.** The results indicate that the eigenpair sensitivity method can accurately identify the parameter damage degree when there is no measurement noise. As the artificial measurement noise level gradually increases, the error of the parameter damage recognition result also gradually increases. When the noise level of 15% is reached, the errors of the parameter damage identification results are within 3%. Thus, the proposed method is robust to noise disturbances in high-damage cases.

### 4.2. Example 2: A Laminated Beam Model of PWS-91 Parallel Cable with 5 Meters (Short Cable)

**Figure 18:**
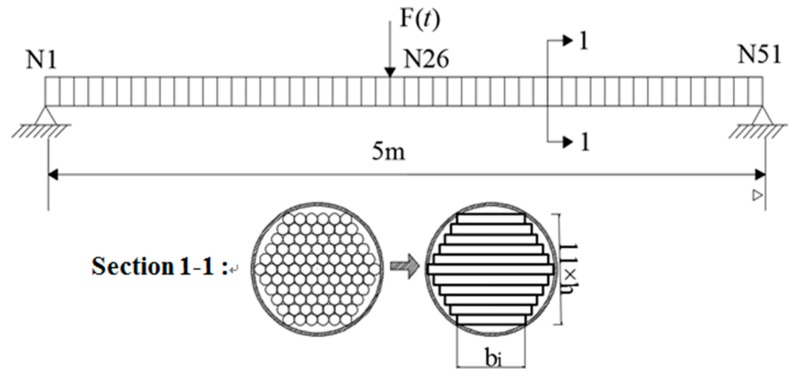
Frictional laminated beam model of PWS-91 parallel cable.

The second example involves a laminated beam model of the PWS-91 parallel cable shown in Figure 18. The length of the cable model is 5 meters, with 50 elements and 51 nodes. The cable is simulated as eleven laminated sections that are 77 millimeters high and 25 to 45 millimeters wide. The physical parameters of the beam model are as follows: mass density *ρ* = 7.9 × 103 kg/m^3^ and Young’s modulus *E* = 195 GPa.

Assuming that the laminated beam is a Euler-Bernoulli beam, its element mass matrix and element stiffness matrix can be expressed as

$$M_{element}=\frac{\rho Al}{420}\left[\begin{array}{cccc} 156 & 22l & 54 & -13l\\ 22l & 4l^{2} & 13l & -3l^{2}\\ 54 & 13l & 156 & -22l\\ -13l & -3l^{2} & -22l & 4l^{2} \end{array}\right]\;,\;K_{element}=\frac{EI}{l^{3}}\left[\begin{array}{cccc} 12 & 6l & -12 & 6l\\ 6l & 4l^{2} & -6l & 2l^{2}\\ -12 & 13l & 12 & -6l\\ 6l & 2l^{2} & -6l & 4l^{2} \end{array}\right]$$

The damping of the laminated beam is assumed to be Rayleigh damping. Referring to the test results of cable damping characteristics in the relevant literature [23,24,25,26,27], the first two modes of the modal damping ratio are set to 0.003.

The reduction in the local bending stiffness of the cable caused by interlayer slip damage appears symmetric or asymmetric in multiple elements located at both the fixed end and external load position. Four different cases are studied in this example, including symmetrical damage in the first slip state, symmetrical damage in all slip states, asymmetrical damage in the second slip state and asymmetrical damage in all slip states. The robustness to measurement noise disturbance is also studied in case 9.

Case 5: Symmetrical damage in the first slip state (short cable)

**Figure 19:**
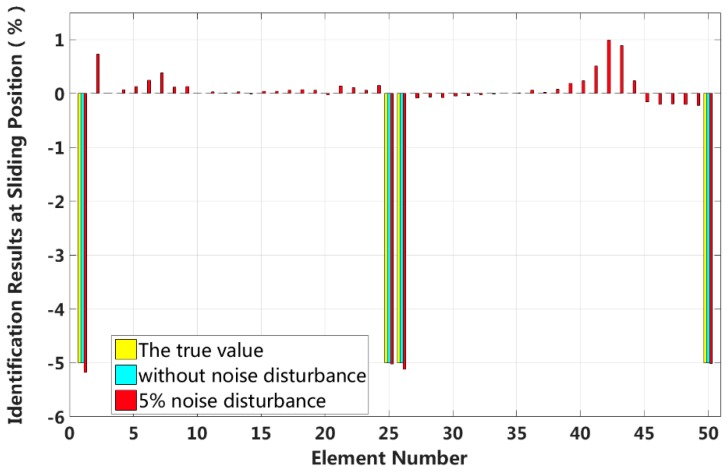
Results of interlayer slip damage identification.

**Figure 20:**
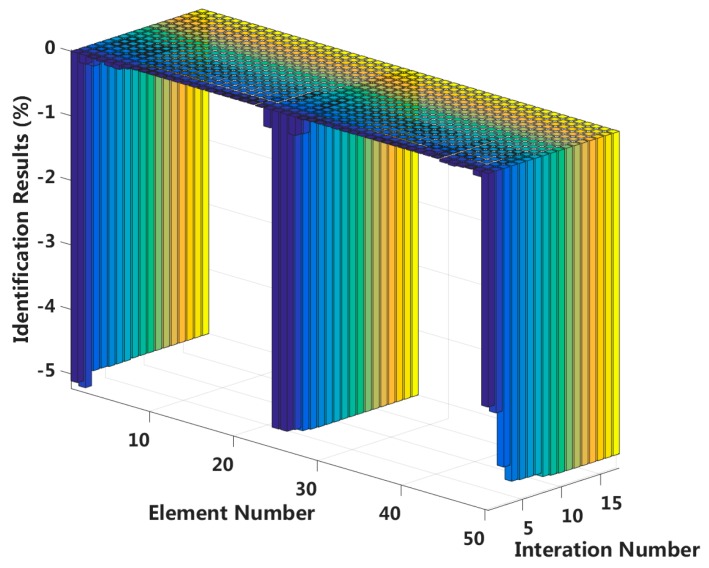
Interlayer slip damage identification results for each iteration.

We assume that a small external load is applied in the middle of the laminated beam and that four elements located at both the fixed end and the middle span enter the first interlayer slip damage state. The identification results of interlayer slip damage are shown in Figure 19, and the results for each iteration are shown in Figure 20. In the case of a 5% level of measurement noise, the stiffness values for the interlayer slip damage elements are −5.18%, −5.02%, −5.11% and −5.01%. Additionally, the stiffness damage identification errors of non-slip elements are within 1%. Therefore, the damage identification results of the proposed method can accurately determine the position and extent of the slip damage in the case of a small applied load.

Case 6: Symmetrical damage in all slip states (short cable)

**Figure 21:**
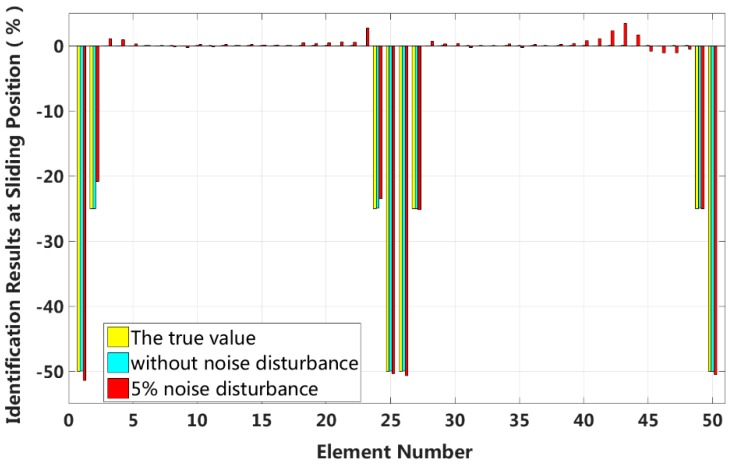
Results of interlayer slip damage identification.

**Figure 22:**
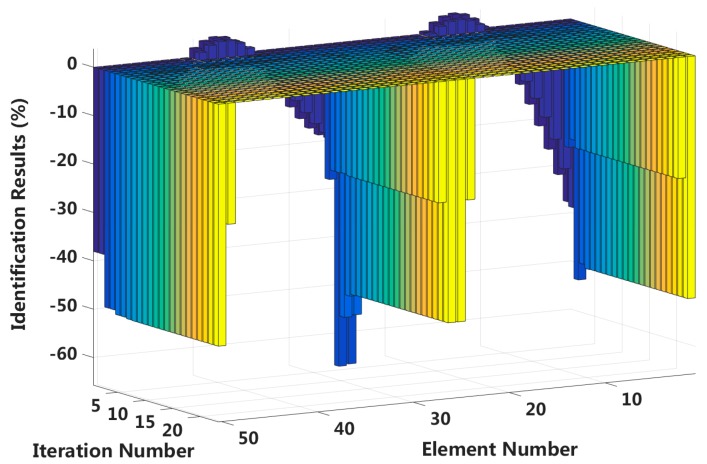
Interlayer slip damage identification results for each iteration.

In this case, we assume that a large external load is applied in the middle of the laminated beam. Four elements located at both the fixed end and the middle span are become the full interlayer slip damage state, and the four elements next to these elements are in the ***j***-th interlayer slip state. The identification results of the interlayer slip damage are shown in Figure 21, and the results for each iteration are shown in Figure 22. In the case of a 5% level of measurement noise, the stiffness changes of the eight interlayer slip damage elements are −51.45%, −20.86%, −23.48%, −50.37%, −50.69%, −25.24%, −25.01% and −50.49%. Additionally, the stiffness damage identification errors of the non-slip elements are less than 2%. This level is lower than the stiffness damage value of the first slip state, and such elements can be easily identified as non-slip elements. This result shows that the proposed method can effectively locate the slip damage elements and accurately identify the interlayer slip damage in complicated conditions involving all interlayer slip damage states.

Case 7: Asymmetrical damage in the second slip state (short cable)

**Figure 23:**
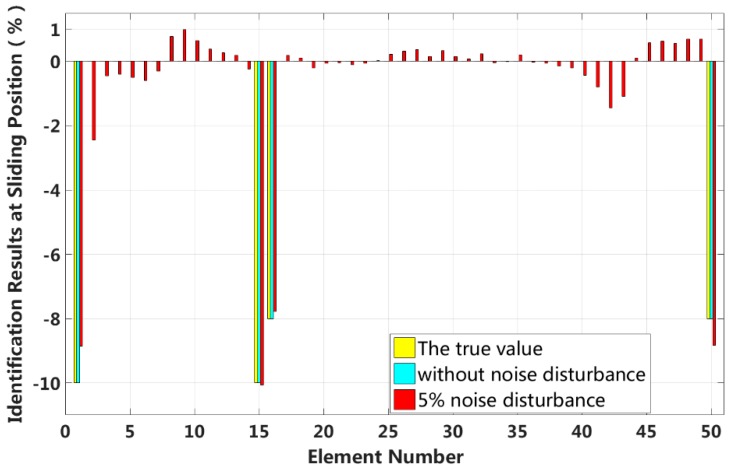
Results of interlayer slip damage identification.

**Figure 24:**
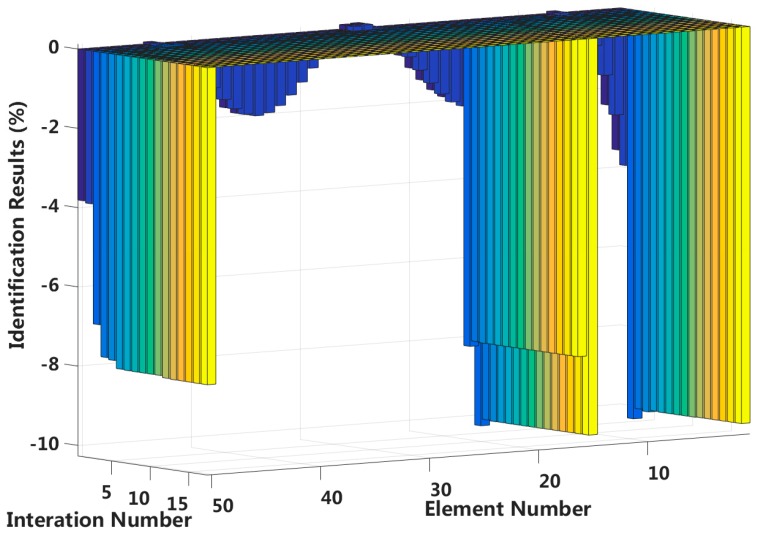
Interlayer slip damage identification results for each iteration.

In this case, we assume that a small external load is applied to the 1/4 span of a laminated beam and that four elements located at both fixed end and the 1/4 span reach 2nd interlayer slip damage state. The identification results of the interlayer slip damage are shown in Figure 23, and the results for each iteration are shown in Figure 24. In the case of a 5% level of measurement noise, the stiffness changes in the interlayer slip damage elements are −8.85%, −10.05%, −7.78% and −8.84%. Additionally, the stiffness damage identification errors of the non-slip elements are less than 3%.

Case 8: Asymmetrical damage in all slip states (short cable)

**Figure 25:**
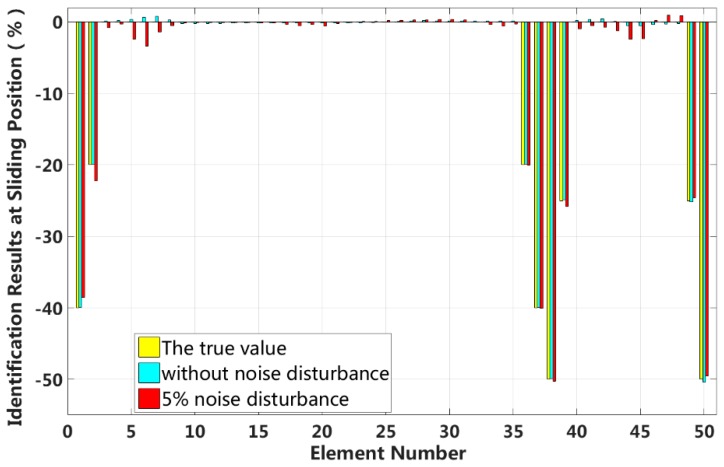
Results of interlayer slip damage identification.

**Figure 26:**
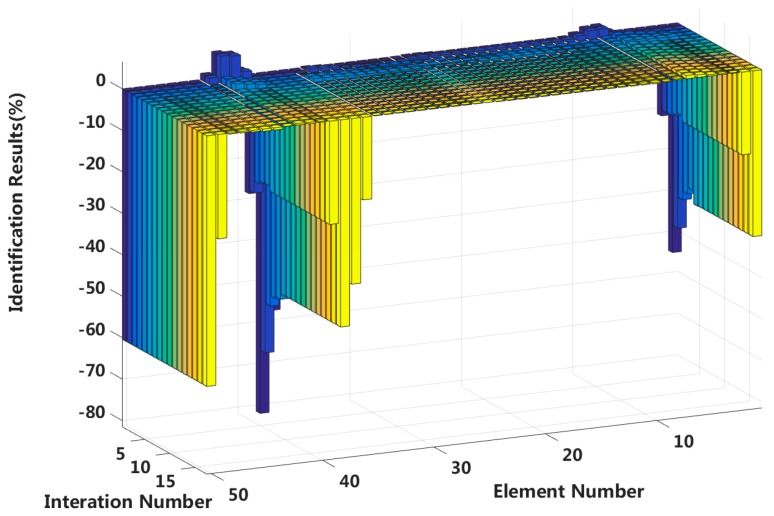
Interlayer slip damage identification results for each iteration.

In this case, we assume that a large external load is applied to the 3/4 span of a laminated beam. Extensive interlayer slip damage occurs in eight elements located at both the fixed end and the applied load position. Figure 25 shows the identification results in the asymmetrical damage state, and Figure 26 shows the results of each iteration. In the case of a 5% level of measurement noise, the stiffness changes of the eight interlayer slip damage elements are −38.52%, −22.28%, −20.01%, −40.04%, −50.23%, −25.81%, −24.58 and −49.55%. The stiffness damage identification errors of non-slip elements are less than 3%, and these elements can be easily identified as non-slip elements because the stiffness damage value of the first slip state is not reached.

Case 9: Robustness to measurement noise disturbances

**Figure 27:**
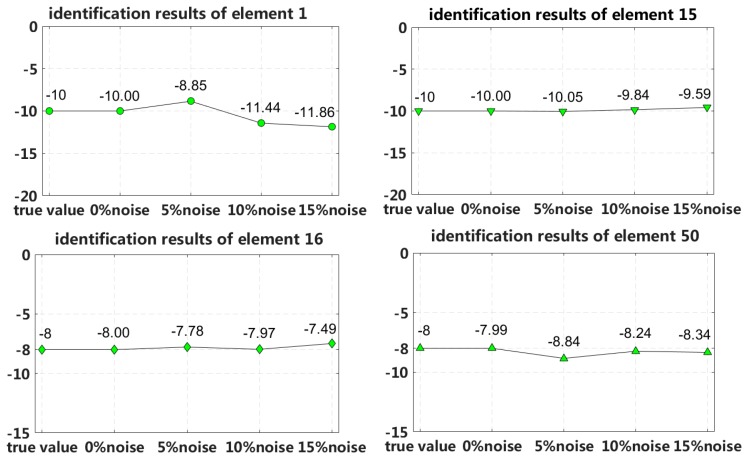
Robustness to measurement noise disturbances.

By comparing the results in cases 5 through 8, we can conclude that the eigenpair sensitivity method is most sensitive to the asymmetrical damage in the second slip state. In other words, interlayer slip damage identification is most sensitive to a measurement noise disturbance when the applied external load is small and asymmetrically applied to a laminated beam. Therefore, case 7 is chosen to study the robustness of the method to noise disturbances. Four different measurement noise levels are studied, and the results are shown in Figure 27.

The proposed method can accurately identify the interlayer slip damage in the case of no measurement noise disturbance. The error of the identification results increases with increasing noise level. The identification errors of interlayer slip damage are within ±2%, even when the noise level reaches 15%. Thus, the proposed method of interlayer slip damage identification is robust to noise disturbances.

### 4.3. Example 3: A Laminated Beam Model of PWS-91 Parallel Cable Length of 30 Meters (Long Cable)

**Figure 28:**
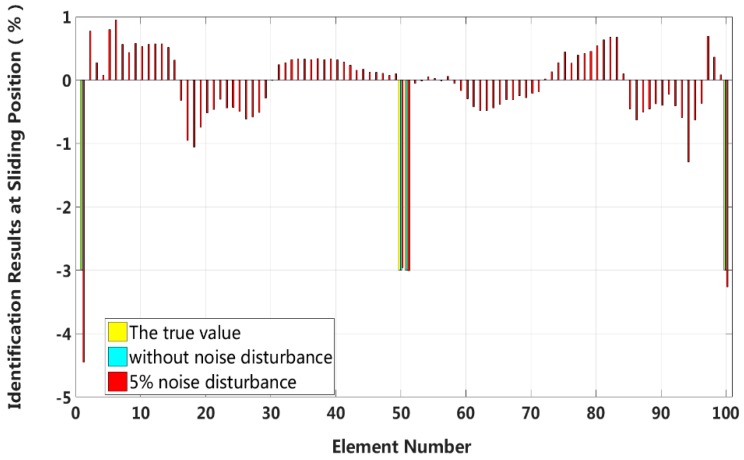
Results of interlayer slip damage identification.

**Figure 29:**
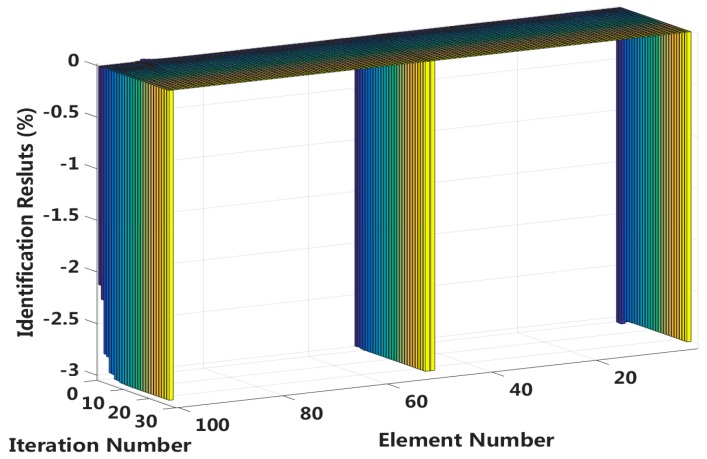
Interlayer slip damage identification results for each iteration.

**Figure 30:**
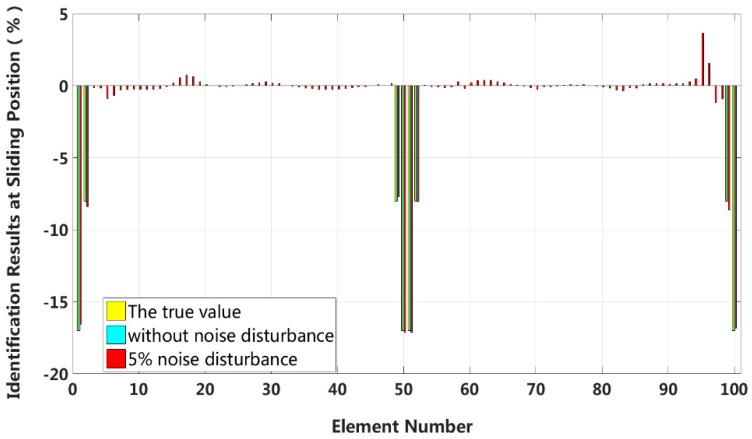
Results of interlayer slip damage identification.

It can be seen in example 2, above, that for the 5 m short cable, the proposed method can accurately identify the stiffness loss caused by the slip damage and has good robustness to the artificial measurement noise. The numerical example 3 increases the length of the structure shown in Figure 18 to 30 meters, and the physical parameters and structural section properties are consistent with example 2. The 30 meters of cable in numerical example 3 are divided into 100 elements and 101 nodes, and only the case where the external load is applied at the mid-span position of the structure is calculated here. The identification results are shown as follows:

Case 10: Symmetrical damage in the first slip state (long cable)

We assume that a small external load is applied at the middle of the laminated beam and that four elements located at both the fixed end and the middle span enter the first interlayer slip damage state. The identification results of interlayer slip damage are shown in Figure 28, and the results of each iteration are shown in Figure 29. In the case of a 5% level of measurement noise, the stiffness values for the interlayer slip damage elements are −4.45%, −2.95%, −3.01% and −3.26%. Additionally, the maximum stiffness damage identification error of non-slip elements is −1.29% in the No.94 element. However, in the case of without noise disturbance, the identification results in all elements are basically the same.

Case 11: Symmetrical damage in all slip states (long cable)

**Figure 31:**
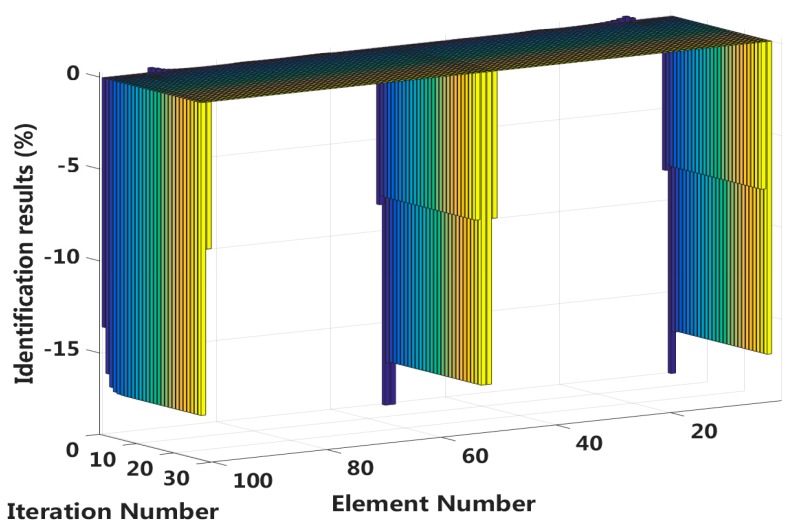
Interlayer slip damage identification results for each iteration.

In this case, we assume that a large external load is applied at the middle of the laminated beam. Four elements located at both the fixed end and the middle span are become the full interlayer slip damage state with 17% stiffness reduction, and the four elements next to these elements are in the ***j***-th interlayer slip state with 8% stiffness reduction. The identification results of the interlayer slip damage are shown in Figure 30, and the results for all iteration are shown in Figure 31. In the case of a 5% level of measurement noise, the stiffness changes of the eight interlayer slip damage elements are −16.57%, −8.38%, −7.71%, −17.14%, −17.14%, −8.06%, −8.62% and −16.83%. Additionally, the maximum stiffness damage identification error of the non-slip elements is 3.64% in No.95 element. This level is lower than the stiffness damage value of the first slip state, and such elements can be easily identified as non-slip elements. 

To verify the feasibility of the proposed method when the cable length and the element length change, Table 2 lists the damage identification results under three different conditions, including cable lengths of 5 m and 30 m, and element lengths of 0.1 m and 0.3 m.

Table 2 shows that for short or long cables, the proposed eigenparameter sensitivity method can accurately identify the stiffness damage caused by interlayer slip. Even in the 5% artificial measurement noise disturbance conditions, the damage identification result is smaller than the true value of the damage degree. However, it can be seen from Table 2 that as the element number of the long cable increases, the number of iterations and the minimum eigenpair order also increase. This means that if the damage location range is changed from 0.3 m to 0.1 m (that is, the element number changes from 100 to 300), the proposed method will significantly increase the computational cost while ensuring the accuracy of damage identification. However, the previous research results indicate that at least the first ten-order eigenparameter data can be successfully acquired using acceleration sensors or computer vision techniques in engineering applications or structural experiments. In addition, these eigenparameter data can be applied to subsequent analysis such as damage identification and model updating of structures effectively [28,29,30,31,32,33]. Moreover, with the maturity and popularity of new technologies such as computer vision measurement technology, the collection of cable eigenparameter data will be increasingly accurate and low cost. So it is practical for the proposed method to be applied to structural experiments and real engineering projects.

## 5. Conclusions

The relative slip between wires in a cable is equivalent to the interlayer slip of a laminated beam. In this study, a laminated beam is simulated as a discrete structural system, and an eigenparameter sensitivity method is used to identify the damage caused by the interlayer slip. The results of different examples show that the proposed method can simultaneously and accurately identify the damage variations in the stiffness and damping parameters of the structure. For complex cases of multiple instances of damage caused by the slip between cables, the proposed method can effectively identify the slip damage elements and quantify the interlayer slip damage. Additionally, the method is robust to measurement noise disturbances. The accuracy of the identification results can be improved with more modal data in the eigenparameter sensitivity matrix when large-scale elements are identified. However, more iterations and calculations will be required. In this paper, currently, theoretical methods and numerical examples have been studied and verified. Subsequent research will further analyze the application of this method with more experimental data or engineering measured data.

## 6. Future Work

The continuation of this research will include the following:

(1) Improve the experimental design of Numerical example 2 and verify the reliability of the proposed method with experimental measured data;
(2) Research on parameter identification and model updating based on experimental measured data to explore noise robustness in the real-life measurements;
(3) Further study the influence of other affective factors of long-span cable structure on the damage identification of interlayer cable slip.

## Figures and Tables

**Table 1:** Error comparison of the damage identification results with and without noise.

Degree of the Noise Disturbance	Stiffness		Damping	
	Result	Relative Error (%)	Result	Relative Error (%)
0%	−30.00	0.00	19.97	0.15
5%	−27.51	8.33	17.28	13.60

**Table 2:** Maximum identification results of structural and element length variation.

Cable Length (m)	Number of Element	Element Length (m)	Iteration Number	Minimum Eigenpair Order Used	Without Noise Disturbance		5% Noise Disturbance	
					Red. (%)	Res. (%)	Red. (%)	Res. (%)
5	50	0.1	21	5	49.9	0.1	20.9	4.1
30	100	0.3	25	7	16.9	0.1	3.6	3.6
30	300	0.1	30	10	50.4	0.4	3.9	3.9

Notes: (1) ”Red.” denotes Reduction, “Res.” denotes Relative error; (2) The data in the table are the damage identification results of the symmetrical all slip state in the case of without or with 5% noise disturbance.
